# Blood flow analysis of retinal neovascularisations in a VLDLR mouse model using contrast-enhanced optical coherence tomography

**DOI:** 10.1364/BOE.574030

**Published:** 2026-01-28

**Authors:** Yash Patel, Bernhard Baumann, Conrad Merkle

**Affiliations:** 1 Center for Medical Physics and Biomedical Engineering, Medical University of Vienna, Währinger Gürtel 18-20/4L, 1090 Vienna, Austria; 2 Institute of Biomedical Physics, Medical University of Innsbruck, Müllerstraße 44, 6020 Innsbruck, Austria

## Abstract

Age-related macular degeneration (AMD) is a major cause of global blindness that affects millions worldwide. Certain forms of AMD cause neovascularisations (NVs), which form retinal-choroidal anastomoses. This disrupts healthy haemodynamic patterns, and early detection and treatment are crucial for preserving vision. Here, we employ a custom-built optical coherence tomography (OCT) imaging system to investigate these NVs in a very low-density lipoprotein receptor (VLDLR) knockout mouse model. Mice were imaged before, during, and after contrast agent injection, aiming to enhance our understanding of the NV haemodynamics. Doppler signal analysis techniques were employed to calculate flow velocities within individual NVs. Flow rates pre- and post-injection were determined based on these velocity measurements. Particle tracking was performed on two NVs for a comparative analysis with the Doppler velocity measurements. Both methods of measuring flow velocities showed good agreement post-contrast injection. The analysis of post-injection flow rates from the NVs revealed diverse behaviours. Some NVs exhibited stable flow rates over time, while others showed signs of instability, with flow rates changing substantially or even changing flow direction at different time points. Additionally, it was observed at multiple time points that flow from certain NVs moved from the choroid to the retina at the same time that other NVs displayed flow in the opposite direction. These observations suggest complex interactions between choroidal and retinal vascular networks in diseases like AMD. Further characterisation using contrast-enhanced Doppler OCT may improve our understanding of neovascular haemodynamics.

## 1. Introduction

Age-related macular degeneration (AMD) is a leading cause of irreversible vision loss and blindness among the elderly worldwide, with almost 200 million people affected globally with AMD, a number expected to reach 288 million by 2040 [1]. Although the neovascular or wet form of AMD (nAMD) accounts for only about 10-15% of all AMD patients, it is responsible for about 80-90% of cases of severe vision loss and blindness associated with AMD [2–4]. The growth of abnormal, leaky blood vessels in the macula results in disruption of central vision of patients, leading to a rapid deterioration in visual function [5–7]. As the average age of the global population is increasing, nAMD poses a growing public health concern, making it essential to better understand it, so that effective strategies for early diagnosis, monitoring, and treatment can be developed.

Understanding retinal blood flow is fundamental for elucidating both physiological and pathological processes in the eye. In a healthy retina, blood flow is regulated to support the metabolic demands of neural and photoreceptor layers, and any form of disruption can compromise visual function [8–12]. Studying blood flow in nAMD is especially important as the disease is characterised by the growth of abnormal, leaky vessels that disrupt normal retinal haemodynamics, leading to oedema, haemorrhage and vision loss [13–16]. Historically, fluorescein angiography (FA) has been the gold standard for imaging and diagnosis of nAMD, providing critical insights into the morphology and leakage characteristics of choroidal neovascularisation (CNV) and other macular neovascular lesions such as type 3 macular neovascularisation (MNV3) and retinal angiomatous proliferation (RAP) [17,18].

Scanning laser ophthalmoscopy (SLO), combined with FA or indocyanine green angiography (ICGA), provides high-resolution imaging of retinal and choroidal vasculature [19]. Widefield SLO systems help visualise peripheral vascular imaging beyond the range of standard imaging modalities [20], making them valuable for evaluating the extent of vascular pathology in AMD. Adaptive optics scanning laser ophthalmoscopy (AO-SLO) combines SLO with optical aberration correction. With a lateral resolution of ∼2 μm, it enables cellular level imaging of individual photoreceptors, retinal pigment epithelial cells and capillaries [21–23]. Laser Doppler holography (LDH) is another technique with high temporal resolution and retinal and choroidal blood flow measuring capabilities [24,25]. LDH has been previously used to detect flow reversals [26], thus showing its capabilities at offering quantitative haemodynamic information through analysis of Doppler frequency shifts.

Nevertheless, FA is inherently limited by its two-dimensional nature, lack of depth resolution, and the intense leakage of dye from lesions which can obscure underlying vascular structures and make it difficult to track smaller and vertically oriented neovascular anastomoses [12,27,28]. SLO based imaging modalities, while offering improved resolution, are fundamentally planar imaging modalities that provide limited information about the depth and structure of the retinal vessels and anastomoses [12,29]. Although AO-SLO provides very high lateral resolution, its typical small field of view of ∼1-2°, complex image acquisition and processing, and primarily planar imaging capabilities [23] limits its ability to assess the three-dimensional extent of NVs. LDH, on the other hand, provides high temporal resolution for quantitative blood flow measurements, but it requires complex spatio-temporal filtering to suppress artefacts from eye motion and lacks intrinsic three-dimensional scanning capabilities [26,30]. As a result, NV studies based on these techniques may make it difficult to correctly estimate the true extent and structural complexity of neovascular networks, particularly during the early stages of the disease.

The advent of optical coherence tomography (OCT) has revolutionised retinal imaging by its non-invasive, high resolution and cross-sectional retinal imaging capabilities [31–35]. OCT angiography (OCTA) is a functional extension of OCT and enables three-dimensional visualisation of retinal and choroidal perfusion based on the intrinsic motion contrast of erythrocytes, allowing for improved detection and characterisation of neovascular lesions [36–38]. OCTA has its own limitations, the visibility of vertically diving or ascending micro-vessels can be compromised by the orientation-dependent backscattering of the red blood cells (RBCs), slow flow rates, and signal attenuation with depth [39,40]. To address these challenges, contrast-enhanced OCT (CE-OCT) using highly scattering agents, such as Intralipid 20%, has been proposed and shown to enhance the signal from otherwise difficult to visualise diving micro-vessels [27,40–42]. Another functional extension of OCT is Doppler optical coherence tomography (Doppler OCT). It enables quantitative measurement of blood flow velocity within retinal vascular networks. By detecting Doppler phase shifts caused by moving erythrocytes, Doppler OCT provides direct, non-invasive assessment of flow dynamics, offering valuable insights into retinal vascular haemodynamics and pathology [43,44].

Despite these advances, there is still a lack of comprehensive, quantitative studies examining blood flow dynamics within retinal neovascularisations, particularly in pre-clinical models that recapitulate human nAMD, such as the VLDLR knockout mouse model [27,45,46]. Most of the previous studies have focused on morphological features, leakage, and qualitative imaging of neovascular lesions, with little to complete lack of quantitative measurement of blood flow velocity, directionality, or heterogeneity within these abnormal vascular networks [17,27,45,47–49]. Existing quantitative velocimetry methods, such as laser Doppler flowmetry and two-photon microscopy, are limited by insufficient depth resolution, restricted field of view, or slow speed to capture the complex, three-dimensional haemodynamics of retinal neovascularisation in-vivo [50–52].

Through this study, we aim to fill this knowledge gap by combining CE-OCT with Doppler OCT to quantitatively analyse blood flow velocity and patterns in retinal neovascularisations in the VLDLR knockout mouse model. By leveraging the enhanced signal provided by an OCT contrast agent, we could overcome some of the limitations of traditional OCT and provide, to our knowledge, the first quantitative assessment of flow dynamics within these NVs in-vivo. With our approach, we offer a more detailed understanding of neovascular haemodynamics, with the potential to reveal new insights into the pathophysiology of MNV3/RAP, and to provide a valuable tool for evaluating therapeutic strategies targeting neovascular flow.

## 2. Methods

### 2.1. Imaging system

A previously described custom-built polarisation-sensitive OCT system [53] was used for imaging mice before, during and after injection of a contrast agent [54]. The spectral-domain OCT ophthalmoscope operated on the central wavelength of 840 nm and a bandwidth of 100 nm (full width at half maximum), system sensitivity of 96 dB, beam diameter (at pupil plane) of 0.5 mm, incident corneal power of 2.85 mW, an A-line rate of 83 kHz, and axial resolution of 5.1 µm in air (∼3.8 µm in tissue with n = 1.35) [53,54].

### 2.2. Experimental protocol

A VLDLR knockout mouse model (n = 6, B6;129S7-*Vldlr*^*tm1Her*^/J, The Jackson Laboratory, Bar Harbor, USA) was used for this study, consisting of 4 male and 2 female mice, aged from 6 to 81 weeks. All imaging sessions were performed under anaesthesia, with either isoflurane or with ketamine/xylazine, as described in our previous study [54]. For isoflurane, mice were induced initially with 4% in oxygen for 4 minutes, then maintained at 2% in oxygen at a flow rate of 1.5-2 L/min while imaging. In cases where mice (n = 2) did not remain adequately anaesthetised with isoflurane, anaesthesia was improved using an intraperitoneal injection of ketamine/xylazine (10 mL/kg body weight) (100 mg/kg ketamine and 6 mg/kg xylazine). The mice were placed on a heating pad throughout the imaging duration to maintain body temperature. The pupils were dilated with one drop per eye of Tropicamide (5 mg/mL, Agepha Pharma s.r.o., Senec, Slovakia) prior to imaging, and artificial eye drops (Oculotect, ALCON Pharma GmbH, Freiburg im Breisgau, Germany) were applied regularly to maintain moist corneas and prevent corneal dehydration during the imaging [54].

Intralipid 20% (3 mL/kg body weight) bolus injection, administered via the teil vein, has been used previously to enhance OCT flow signals [54,55]. We use it here as a highly scattering OCT contrast agent to enhance the signals in vessels most affected by OCT artefacts [39,56], in particular NV anastomoses, which orient more parallel to the OCT beam [54]. We used a 3D angiography scan protocol to generate an angiogram for a 1 mm x 1 mm scanning area centred on the optic nerve head. This protocol included acquiring data with a scan density of 500 A-scans per B-scan, along with five repeated B-scans at each of the 400 slow-axis positions, which took approximately 16 seconds to capture. This protocol was used to obtain 3D data sets, with one pre-injection dataset, and up to three post-injection datasets, obtained over the course of 12 minutes after contrast injection for each mouse.

During the initial passage of the contrast agent, we also employed a Dynamic-Contrast OCT (DyC-OCT) scan protocol [42] that involved repeated sampling of the same B-scan cross-section (interscan time 7.7 ms, 2000 time-points) to capture neovascularisation dynamics. The DyC-OCT scan was also performed over a 1 mm range along the fast axis, ensuring comprehensive coverage in the axial (z), lateral (x), and temporal (t) dimensions. All animal protocols were approved by the ethics committee of the Medical University of Vienna and the Austrian Ministry of Education, Science, and Research (BMBWF/66.009/0272-V/3b/2019).

### 2.3. Post processing

In accordance with our previous studies [45,54,57], we utilised the strong intensity signal from the retinal pigment epithelium (RPE) in the cross-polarised OCT channel for frame alignment and retinal flattening purposes [45]. The angiogram signal was derived by applying the complex subtraction OCTA method to the five repeated B-scans for all time-points [58,59]. In order to produce high quality angiogram masks, this data was subjected to a spatial filtering process in two steps to enhance the vessel signals and mitigate background noise.

Initially, a 3 × 3 averaging filter was applied to the angiogram B-scans (Angio3) to smooth out high-frequency noise. A binarisation threshold was then determined after applying a more aggressive 10 × 10 averaging filter (Angio10) to minimise the effects of high-intensity, small-scale outliers. For each Angio10 B-scan, mean values were calculated across the fast-axis (x) to generate a 1D depth profile. The maximum value across all depth profiles was selected as the reference value for that angiogram C-scan. This approach ensured that a single global threshold was selected for each scan time point.

To generate masks of the vasculature, the Angio3 data was then subjected to binary thresholding using 9% of the above reference values from the previous step. In one angiogram volume, this threshold was adjusted to 12% of the reference to ensure a similar angiogram mask quality. This thresholding technique isolated the vessel signals from the background, enhancing the contrast between the vessels and the surrounding tissue noise in the angiogram B-scans. These binary masks were then used for further processing steps. All analyses were conducted using MATLAB (R2024b, MathWorks, Natick, MA, USA).

### 2.4. Velocimetry

The study offered an opportunity to compare two different velocimetry techniques, particle tracking and Doppler flow, using the same neovascularisations. The two vessels were selected based on several criteria to ensure suitability for both velocimetry techniques. First, the vessels needed to be located within the usually avascular outer nuclear layer (ONL) to ensure classification as NVs. Second, vessels needed to be clearly visible in the DyC-OCT intensity images with sufficient vertical orientation to the scanning beam for Doppler velocity measurements. Finally, the vessels had to be parallel to the imaging plane and visible for a sufficient length within a single B-scan to enable continuous tracking along the vessel length for particle tracking velocimetry. The region of interest (ROI) encompassing these abnormal vessels were segmented from the intensity B-scan images manually before applying each of the two techniques.

#### 2.4.1. Particle tracking velocimetry

**Fig. 1.**
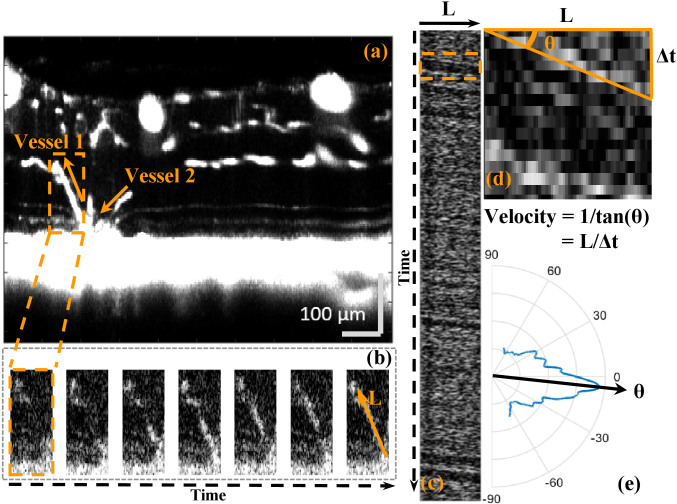
OCT velocimetry performed using a particle tracking technique along NV vessel segments. (a) Angiogram signal used for segmentation of the vessels. (b) Tracking of the intensity signal of vessel 1 through time. The orange arrow shows the selected vessel segment for particle tracking. (c) Time stack of the tracked vessel 1 segment. (d) Zoomed in view of the streak pattern shown in (c). (e) Distribution of the calculated angle values using Radon transform, and the peak of this distribution is taken as the dominant angle θ.

One set of velocity measurements was obtained by employing a particle tracking method along manually identified vessel segments, leveraging methods typically used for fluorescence-based blood velocimetry [60]. The DyC-OCT B-scan (Fig. 1(a)) was positioned to scan the neovascular lesions located approximately 450 µm superior to the optic nerve head.

The DyC-OCT intensity images captured by our system enabled tracking of the in-plane red blood cell scattering patterns as they moved through the vessels (Fig. 1(b)). For each vessel segment, a series of intensity profiles were obtained along the vessel, enabling the tracking of blood and contrast agent movement over time within the segmented ROI. By constructing a time stack of the intensity signal along the tracked vessel segments, the dominant flow streak angle (*θ*) could be extracted using Radon transform (Fig. 1(c)–(e)) [60,61]. The angle is directly related to the quantitative velocity, which is calculated as:

$$V_{PT}=\frac{1}{tan(\theta )}=\frac{L}{\Delta t}$$

Where $V_{PT}$ is the particle tracking velocity along the vessel segment, *L* is the length of the vessel segment and Δt is the time that it takes for a particle to travel the distance of that segment. The Radon transform for particle tracking was performed with a time window of 100 B-scans, which corresponds to approximately 0.77 seconds, given that the time between B-scans is 7.7 ms. This time window was then shifted in steps of 1 B-scan, so that consecutive time windows then overlapped by 99 B-scans (all but one B-scan). This sliding window approach provided velocity measurements with smoother temporal sampling.

#### 2.4.2. Doppler velocimetry

The Doppler phase difference was calculated by applying previously established methods [62–65] to the DyC-OCT data set. Phase difference bias introduced by the scanning galvos was corrected by subtracting the weighted average of the phase difference within the retinal pigment epithelium (RPE) layer [58,66].

To correct the impact of the residual static background Doppler phase difference bias in the surrounding tissue, additional Doppler phase difference correction was implemented. The mask encompassing the vascular ROI was dilated by one pixel for each B-scan to ensure complete vessel coverage and inclusion of the flow missed by the segmentation in the peripheral region of the vessels. For each depth (z), a region of interest on either side of the dilated vessel ROI extending 15 pixels in the lateral (x) and temporal (t) dimensions was used to identify the local Doppler phase difference bias. The mean noise within this ROI was calculated for each depth position and then subtracted from the Doppler phase difference values of the pre-dilated NV mask. Temporal averaging was then performed across the five repeated measurements. We refer to this corrected Doppler phase difference as $\Delta \phi_{corr}$ .

The axial flow velocity $\left(V_{axial}\right)$ was calculated for each B-scan, utilising the corrected Doppler phase difference as:

$$V_{axial}(z,x,t)=\frac{\lambda }{4\pi nT}[\Delta \phi_{corr}(z,x,t)]$$

where λ is the central wavelength, *n* is the refractive index, and *T* is the time duration between two adjacent A-scans. This method of velocity calculation provided directional axial velocity, where blood flow in the direction of the scanning axis resulted in positive velocity values, while against the axis resulted in negative velocity values. To quantify the net velocity of the entire vessel, the average velocity over each vascular ROI was computed. This approach enabled more accurate quantification of blood flow velocities in the selected NVs.

To calculate the longitudinal component of the axial velocity along the direction of the vessels, a correction has to be applied based on the angle of the vessel. In this study, this vessel angle was measured from the manual segmentation of the vessels. The longitudinal velocity was then calculated as:

$$V_{D}=\frac{V_{axial}}{cos\alpha }$$

where $V_{D}$ is longitudinal Doppler velocity and α is the angle of the vessel with respect to the incident beam. The average absolute velocity was calculated over the vascular ROIs, and the changes in velocity were tracked pre- and post-contrast injection.

### 2.5. Doppler flowmetry

The post injection 3D angiogram and Doppler data was used to manually identify NV anastomoses and control vessels in the en face plane of the outer nuclear layer to calculate flow rates pre- and post-contrast injection. The outer nuclear layer was selected because it is a low intensity avascular layer, making the identification of abnormal vessels significantly easier. A custom LabVIEW (2023 Q3, National Instruments, Austin, TX, USA) GUI was developed to visualise the B-scan angiogram and axial velocity en face maps side by side, enabling precise NV identification and ROI definition. NVs were initially identified in post-contrast B-scan angiograms, and the centre of each NV was manually annotated to guide the placement in 3D space. This process was performed for all post-injection time points, where NVs were clearly visible. Common NVs that appeared across all post-injection time points were identified and chosen for the study. These same NVs were then located in the pre-injection time point data based on their position relative to anatomical landmark features, despite the NVs being harder to visualise without the contrast agent. We used the pre-injection data itself to create vessel masks after identifying vessels based on these landmarks, rather than using image registration from post-injection time points to better reflect the way that these Doppler signals would be measured without a contrast agent. The control vessels were selected from diving or ascending vascular branches that were measured in between vascular layers.

Similar to Doppler velocimetry, the Doppler phase difference was calculated and corrected for each vessel ROI identified from the 3D angiography scan protocol. While the 3D data has dimensions (z, x, y) rather than (z, x, t) for the DyC-OCT scans described above, the same methods for correcting the local Doppler phase bias were used with the substitution of the slow axis (y) dimension for the temporal dimension (t). For each NV, the depth plane providing the clearest vessel cross-section near the middle of the NV was selected for flow rate calculation, as the flow rates near the edges of the NV could vary due to the merging with the choroidal and retinal vascular networks. This ensured accurate flow measurements for each individual NV, as different NVs were located at different depths within the retina and had different lengths based on their connection in the retinal network. The flow rate (Q) through the en face plane was then calculated as:

$$Q=\iint_{ROI}v_{axial}(z,x,y)\text{d}x\text{d}y$$

The integration of the axial velocities over a window encompassing the vessel of interest in the en face plane yields the total flow rate of that vessel as per the methods established by Srinivasan et al. [66].

The analysis was divided into pre-injection and post-injection periods to evaluate the method agreement under different signal intensity conditions. The pre- and post-injection period was derived from the analysis of the mean angiogram intensity. This division allowed assessment of measurement agreement both in baseline conditions and during signal enhancement.

Net flow into the choroid, and total flow into and out of choroid, was calculated for the post-injection periods for all the mice included in the study. The average flow directionality was then calculated as average net flow divided by the average total flow of all the NVs, averaged across all timepoints for individual mouse.

While both particle tracking and Doppler velocimetry were employed to measure the blood flow velocities from DyC-OCT scans, these scans are inherently restricted to a given plane and thus cannot provide volumetric flow rate measurements. Therefore, only 3D Doppler data was used for flow rate calculations.

### 2.6. Correlation analysis

To assess the reliability and agreement between the two velocimetry methods, particle tracking and Doppler velocimetry, the correlation coefficient was calculated for the DyC-OCT data. Because particle tracking measurements need to be performed over a larger time window than Doppler OCT, temporal alignment was necessary for direct comparison of the two techniques. A moving average over the same time window of 0.77 seconds was applied to the Doppler OCT data (Doppler average) to match the temporal window used for the particle tracking measurements and provide a fair comparison. This resulted in Doppler OCT measurements match the particle tracking sampling parameters.

## 3. Results

We quantitatively analysed blood flow velocities and flow directionalities in the NVs across all 6 VLDLR knockout mice with the help of CE-OCT and two velocimetry techniques.

### 3.1. Velocity comparison and analysis

**Fig. 2.**
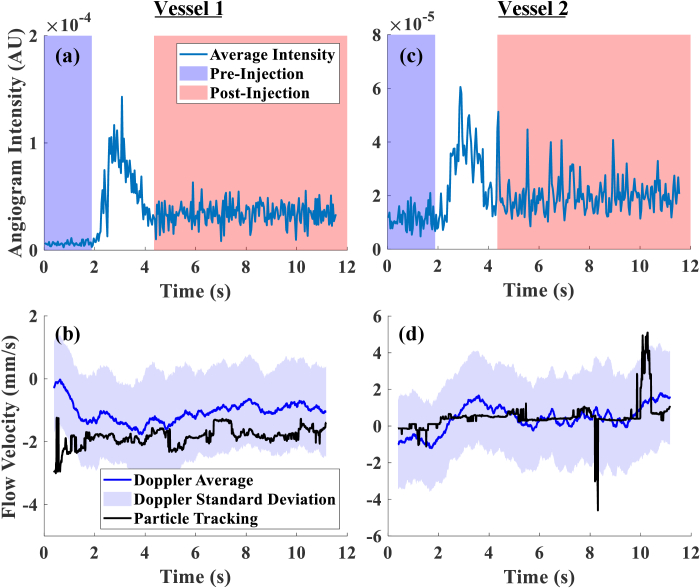
OCT velocimetry performed on the two NV vessels shown in Fig. 1(a). (a) (c) Angiogram intensities measured during the injection with the DyC-OCT protocol at vessels 1 and 2 respectively. (b) (d) Calculated Doppler OCT flow velocities (mm/s), corrected for the angle of the vessel, and the velocities measured using the particle tracking method at vessels 1 and 2 respectively. Negative values indicate the direction of flow going from the choroid into the retina.

Two diving NV segments were selected from the DyC-OCT intensity data from Mouse 1 (Fig. 1(a)). Vessel diameters were measured from the DyC-OCT angiograms and corrected for the vessel angle relative to the imaging plane. The angles were measured as 0.67 and 1.07 radians for Vessel 1 and Vessel 2 respectively. Vessel 1 had a diameter of approximately 28-32 µm, while Vessel 2 was smaller at approximately 13-20 µm. Blood flow velocities were measured in two NV vessel segments using particle tracking and Doppler velocimetry methods during the passage of a contrast agent. Velocities measured in vessels 1 and 2 using the particle tracking method before contrast agent injection were -2.22 ± 0.38 mm/s and -0.11 ± 0.22 mm/s, and after contrast injection were -1.76 ± 0.23 mm/s and 0.66 ± 0.89 mm/s, respectively. In comparison, the average velocities measured using the Doppler OCT velocimetry approach were -0.76 ± 0.50 mm/s and -0.82 ± 0.19 mm/s pre-contrast, which then shifted to -1.02 ± 0.21 mm/s and 0.60 ± 0.49 mm/s post-contrast. While the particle tracking method yielded slightly higher average velocities (Fig. 2(b), d), the two techniques demonstrated good agreement in their post-contrast velocity measurements and followed similar trends over time.

Further analysis of the angiogram data revealed interesting patterns. As shown in Fig. 2(a) and 2(c), the angiogram signal intensity peaked when the contrast agent entered the NVs and then gradually decreased but remained at a higher baseline level than before the injection. This indicates the contrast agent successfully enhanced the visibility of the NVs. Interestingly, post contrast injection, the larger NV vessel 1 did not exhibit substantial changes in velocity over time (Fig. 2(b)), whereas the smaller and more difficult to visualise vessel 2 showed more dynamic velocity changes in response to the tracer arrival (Fig. 2(d)). Specifically, the Doppler velocity of vessel 2 changed with the tracer arrival but then did not return to the pre-injection baseline (Fig. 2(d)) as quickly as its angiogram signal (Fig. 2(c)).

### 3.2. Correlation analysis

**Fig. 3.**
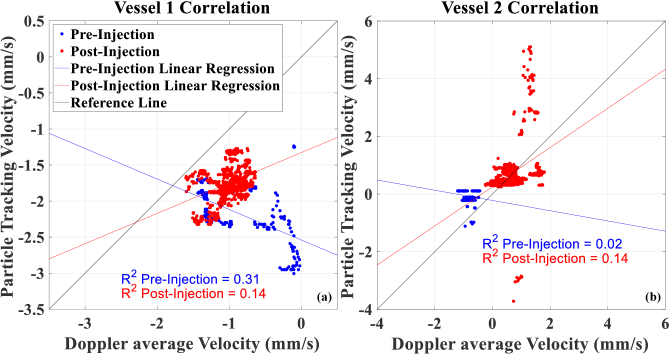
Correlation graph between velocity measurements by Doppler OCT and particle tracking method, before and after injection of a contrast agent for Vessel 1 (a) and Vessel 2 (b) as shown in Fig. 1.

Comparison between particle tracking and Doppler methods demonstrated varying levels of agreement depending on contrast agent presence. Figure 3 presents the correlation graph between Doppler average and particle tracking velocimetry for Vessels 1 and 2 (Fig. 1(a)). For the larger NV vessel 1, the pre-injection measurements showed a negative correlation of -0.56 (R^2^ = 0.31) between the two measurement techniques (Fig. 3(a)). However, following contrast injection, this relationship reversed with a positive correlation coefficient of 0.37 (R^2^ = 0.14).

Similarly, for the smaller NV-Vessel 2, pre-injection measurements showed a slight negative correlation coefficient of -0.15 (R^2^ = 0.02), which then transitioned to a positive correlation coefficient of 0.38 (R^2^ = 0.14) (Fig. 3(b)). This vessel showed more outlying particle tracking velocity measurements in the range of ±2 to ±5 mm/s.

### 3.3. Doppler flow analysis

**Fig. 4.**
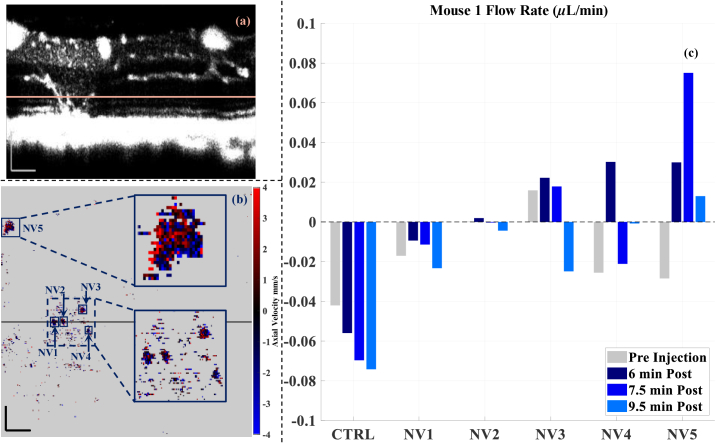
NV flow quantification at different time points for Mouse 1. Angiogram B-scan slice (a) and en face Doppler velocity map (b) from 7.5 minutes post-injection time point. The location of B-scan slice (a) corresponds to the black line in (b), and the location of en face Doppler velocity map (b) corresponds to the pink line in (a). The dark blue boxes (b) show the NVs of interest. Note that the en face Doppler velocimetry map (b) here is captured from uncorrected phase data for visualisation purposes only, and the measurement of the reported flow values for each NV (c) used optimised depth locations with proper noise correction as described in **section 2.4.2**. (c) Flow rates in the different NVs and the control vessel (CTRL). CTRL was acquired from a different plane (not shown). All scalebars are 100 µm. Negative values indicate the direction of flow going from the choroid into the retina.

Figure 4 presents the results from the 3D scans measured pre- and poste-injection for Mouse 1. The control vessel exhibited a relatively stable flow rate over time with a slight continuous increase observed after the contrast agent injection (Fig. 4(c)). Some NVs exhibited relatively stable flow rates post-injection, while others showed unstable flow rates that varied throughout the observation period.

NV1 and NV3 in Fig. 4(b) correspond respectively to Vessel 1 and Vessel 2 in Fig. 1(a). NV1 exhibited flow originating from the choroid and moving into the retina, both before and after the injection, as also observed in Fig. 2(b). In contrast, NV3, NV4 and NV5 switched their flow direction at some point following the contrast injection, a trend also seen in Fig. 2s(d). NV2 on the other hand exhibited very low to no flow through time.

**Fig. 5.**
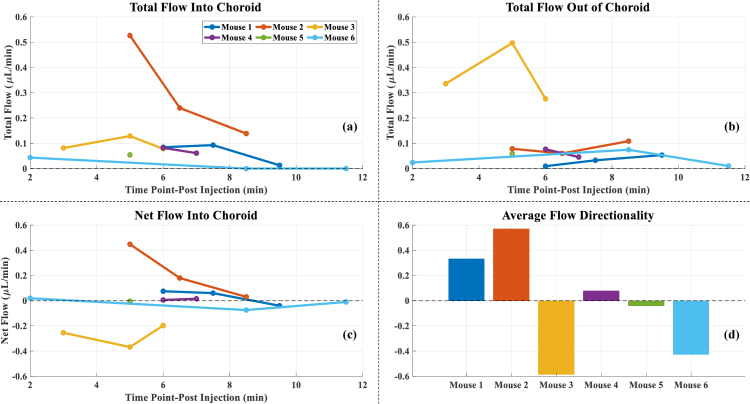
(a) (b) (c) Total flow into and out of choroid, and net flow into choroid respectively for all the mice across different time points. (d) Average flow directionality. Negative values indicate the direction of flow going from the choroid into the retina.

Figure 5 presents different retinal-choroidal flow dynamics in different mice at multiple timepoints post-injection. Figure 5(a) and 5(b) show the total flow into and out of the choroid, with Mouse 2 exhibiting higher flow into choroid (peaking at ∼0.53 µL/min at 5 minutes post-injection) while Mouse 3 exhibiting higher flow out of choroid (peaking at ∼0.5 µL/min at 5 minutes post-injection) compared to other mice. Mice 1, 4, 5 and 6 showed relatively stable total flow values, averaging below 0.1 µL/min over the scanning period. Figure 5(c) shows the net flow into the choroid at different time points, with individual variations observed between mice. Mice 1, 2 and 4 exhibited neutral to positive net flow (indicating flow into the choroid). Mouse 6 initially exhibited a net flow into the choroid, but over time this direction reversed, resulting into net flow out of the choroid. Mouse 3 exhibited predominantly flow out of choroid.

Figure 5(d) shows the average flow directionality across all time points for each mouse. Mice 1 and 2 exhibited positive average flow directionality (0.33 and 0.57 respectively), suggesting predominantly flow into the choroid. Mice 3 and 6 on the other hand exhibited negative average flow directionality (-0.59 and -0.43 respectively), suggesting predominantly flow out of the choroid. Mice 4 and 5 exhibited average flow directionality closer to zero (0.08 and -0.04 respectively), suggesting more balanced or bidirectional flow. In total, 38 NVs were investigated across all 6 VLDLR knockout mice. Of these, 17 NVs (45%) exhibited a reversal in flow direction at some point post-contrast injection, while the remaining NVs maintained a stable flow throughout the scanning period.

## 4. Discussion

Through this study we gained insights into the complex haemodynamics of neovascularisations in VLDLR knockout model mice. We observed aspects of blood flow through these irregular vessels which have not been shown previously. The results derived from this study indicate that retinal-choroidal anastomoses exhibit dynamic behaviour and flow patterns that vary significantly between animals and even between individual NVs within the same eye. Such complex flow behaviour shows NVs have the potential to create abnormal blood flow patterns that might contribute to the pathophysiology of the neovascular AMD, by disrupting the normally separate retinal and choroidal vascular networks. Studies have shown that these abnormal vessels develop functional lumens, and the VLDLR knockout mice carry on to have most of these NVs throughout their life, which may lead to longitudinal impacts [46,67–69].

### 4.1. Blood flow velocity and blood flow dynamics in neovascularisations

While the literature supports the perfusion and remodelling of neovascular complexes [45,48,49,70], quantitative analysis of blood flow velocity and in vivo blood flow dynamics within these individual NVs is not reported to the best of our best knowledge. Previous studies using OCT and FA, in both animal models and patients, have primarily focused on the detection and distribution of the NVs, and not quantifying velocity and flow dynamics. Augustin et al. [45] studied the VLDLR knockout mice using multi-functional OCT and OCTA, and confirmed the presence of abnormal vessels extending from the deep retinal plexus into the subretinal space. Studies in patients with type-3 macular neovascularisation (MNV3) and RAP have been used to characterise the presence of vascular complexes, shunting, and anastomoses [48,49,70], but quantitative assessment of flow in these NVs has been largely limited to qualitative analysis and vessel visualisation.

In a previous study done by Axer-Siegel et al. [48], they demonstrated in newly diagnosed CNV patients, that in cases with both retinal arterial and venous anastomoses present, the typical blood filling sequence was from the retinal arteriole into the neovascular lesion, and then out into the retinal venule. In some cases, only with venous anastomoses, the flow was observed to move from the choroid into the CNV, and then drain into the retinal vein, suggesting specific patterns of vascular connection could result in variable flow directionality. Ravera et al. [49] specifically investigated the presence of shunting in RAP patients using multimodal imaging. Using FA, they observed that blood flow shunting to the RAP lesion was observed in 56% of RAP patients, while no such shunting was observed in any other type of CNV patients. This shunting represents a direct arterial supply to the lesion, reflecting a complex remodelling of vessels. They highlighted that shunting, even though not seen in 100% of cases, is a distinctive feature of RAP in humans.

Decorrelation-based OCT and neural network approaches have helped enable the extraction of flow velocity information from OCT time-series data in retinal microvasculature [56]. Another recent study, by Tanaka et al. [71], developed a programme to analyse blood flow velocity in neovascular AMD using the OCTA variable interscan time analysis (VISTA) [72] approach. However, their measurements do not correspond to absolute blood flow velocity, but rather to a relative flow index that allowed for a comparison between the vessels within the same imaging session. This highlights that while there is interest in quantitative analysis of the blood flow parameters in retinal vessels and NVs, there still are challenges.

Traditional OCTA helps visualise chorioretinal vasculature, but its signal depends on the orientation of vessels relative to the scanning light [39,73], making vessels parallel to the scanning axis less visible [56], and NVs are usually parallel to the scanning beam. The reduced visibility of such diving vessels arises due to the orientation-dependent backscattering properties of RBCs, which are considered as the intrinsic source of OCTA signal. In micro-vessels, RBCs tend to elongate along the vessel axis [39], thus effectively reducing the cross-sectional area for the reflection of the incident beam in these diving vessels. In contrast, the vessels perpendicular to the scanning beam present a larger cross-sectional area, resulting in stronger back reflection. As a result, the diving vessels are more likely to fall below the detection threshold in OCTA.

Intralipid 20% has been shown to improve OCT signal intensity [41,42,54,55]. We observed poor agreement between the two velocimetry techniques before the contrast injection. This was evident by the negative correlation coefficient values (-0.56 and -0.15 for Vessels 1 and 2 respectively). However, the contrast injection administration increased the scattering within these diving vessels and improved the visibility of them. We observed changes in the measured Doppler and particle tracking velocities as a result of the contrast agent, which led to a substantial improvement in the agreement of the velocimetry techniques, as made evident by the positive correlation coefficient values (0.37 and 0.38 for Vessels 1 and 2 respectively). This might suggest that the pre-injection measurements are dominated by noise and artifacts, while the post-injection measurements record the haemodynamics more accurately. This emphasises the use of the exogenous contrast agents for reliable quantitative flow measurement in difficult to visualise vascular structures like NVs, where the poor signal quality often makes the measurements less reliable. Despite their abnormal morphology, the NVs (selected from the DyC scans) showed short-term stability of blood flow velocities for the duration of ∼11 seconds of the DyC OCT scans.

Accurate pre-injection NV segmentation was difficult, even within the outer nuclear layer (ONL), where segmentation should be more straightforward, due to the lack of OCT signal from the smaller diving vessels. This in turn compromised the Doppler OCT flow measurements during the pre-injection time period (Fig. 2(b), d) (Fig. 4(c)). The post-injection boost in contrast of NVs from the surrounding tissue enhanced the visibility of these abnormal vessels, helping with more precise NV segmentation and thus making Doppler OCT flow measurements more reliable (Fig. 2(b), d) (Fig. 4(c)). The pre-injection NV detection challenge limits longitudinal NV flow dynamics monitoring without contrast injection, as the calculated flow values may be unreliable without the enhanced signal intensity. While such experiments could be helpful in determining whether flow direction changes are indeed caused by contrast-enhancement or part of natural temporal fluctuations, the lower signal quality would make it difficult to obtain reliable flow measurements.

Our study provides a quantitative baseline for the neovascular flow in this disease mouse model, which previously has been difficult to analyse due to the limitations of traditional ophthalmic imaging techniques. The control vessels exhibited stable flow rates over time, which made sense considering their integration into the more regulated portion of the retinal vascular network, with reliable connections from retinal arteries and veins. NVs on the other hand, exhibited variable flow, with some showing flow reversals at some time point post-injection. We hypothesise that this instability arises from the connections formed by these NVs between the choroidal and retinal vascular networks, which may demonstrate opposing or unsynchronised pressure differentials and unreliable flow patterns. We observed heterogeneous flow patterns of not only across animals but even between neighbouring NVs within the same NV cluster of an eye. We observed a full spectrum of behaviours ranging from blood flow going predominantly from retina to choroid, to blood flow going predominantly from choroid to retina. Such variable directionality has not been documented previously in neovascular model mice and suggests that these NVs may connect randomly between the choroid and inner retina, rather than preferentially to arterioles or venules. The resulting connections create a complex network of vessels with multidirectional flow, determined by the point of connection to the choroidal and retinal vascular network, and local pressure gradients. Direct quantitative measurements of flow in NVs using techniques such as contrast-enhanced OCT are valuable for understanding the extent of flow redistributions, and therefore impacts to nutrients and waste distribution, and disease progression. Combining this technique with visible light OCT could provide valuable context on oxygen saturation [74–76] that would improve our ability to interpret the impacts that flow through NVs have on retinal tissue.

### 4.2. Comparing different velocimetry techniques

The study provided an opportunity to compare two velocimetry techniques, particle tracking and Doppler flow, on the same vessels. Although both approaches utilise the same OCT data, the source of information is different, which allows a robust comparison of the two methods that’s also temporally co-registered. Vessel 1 Doppler velocimetry measurements showed slower overall velocity compared to particle tracking (Fig. 2(b)). This difference likely stems from the fundamental difference in the measurement principles between the two techniques. Our Doppler implementation provides an average flow value across the entire vessel cross-section, while the particle tracking captures movement at the centre of the vessel where the fast-moving particles are most visible. We believe this difference in velocity measurements might reflect the basic principles of fluid-dynamics in blood vessels, where a parabolic profile is generally observed, resulting in peak velocities at the centre due to the laminar profile. For Vessel 2, which was smaller in diameter, the Doppler velocity was closer to the particle tracking velocity (Fig. 2(d)). We think this could be because, in smaller vessels, the velocity profile is more blunted [77,78], and the difference between the central velocity and the average cross-sectional velocity is reduced [79]. As a very small diving vessel (Fig. 1(a)), Vessel 2 was not clearly visible without contrast enhancement. The pre-injection particle tracking velocity values oscillated very close to zero (Fig. 2(d)), suggesting noise-dominated measurements. Only after contrast injection did the flow direction and velocity become clear and reliably measurable. The apparent flow direction change in Vessel 2 before and after injection (Fig. 2(d), Fig. 3(b)) could therefore be attributed to the transition from noise-dominated signal to more reliable signal measurements, instead of true physiological flow reversal. As a result, the Doppler and particle tracking velocity measurements converge better post-injection for Vessel 2 than Vessel 1. It should be noted that these NVs do not connect to major retinal arteries or veins, and rather connect to the capillary network on the retinal side. This makes it difficult to categorise them as arterial or venous.

In addition, transient low-scattering streaks were observed in the particle tracking measurements, potentially due to the displacement of passage of red blood cells by other blood components [80,81], which could also suppress the Doppler signals, leading to lower average Doppler velocities. Conversely, these low intensity streaks provide strong contrast for particle tracking measurements and do not impact the measured velocity. The general agreement post-injection between the two methods, and the consistent improvement in the correlation coefficients across both the vessels after introducing a contrast agent, suggests that contrast administration can improve the reliability of both techniques and bring them into closer agreement. Correlation between the two methods remains somewhat weak, potentially due to the above differences in the measurement principles, so care should be taken when interpreting the results measured by each type of velocity measurement with regard to the ROI used. Nevertheless, both methods have demonstrated that they can be used to measure velocity in historically challenging vascular structures such as NVs with the aid of a contrast agent.

### 4.3. Isolated vessel

During analysis of one of the timepoints from Mouse 1, we identified a vessel (NV2, visible between Vessels 1 and 2 in Fig. 1) that consistently showed minimal to no flow across all time points (Fig. 4). Upon further investigation, we found that the vessel was anatomically isolated and did not connect to any functional vascular network on the side distal to the lesion, thus effectively making it an isolated vessel originating from the choroid. This observation highlights the utility of our flow quantification method in distinguishing between perfused and non-perfused NVs, and suggests that such approaches may assist in identifying disconnected or non-functional NV anastomoses. A longitudinal study across the development of these NVs in the VLDLR knockout model mice could provide more information on the temporal evolution of these neovascular networks.

### 4.4. Effects of Intralipid on the measured signals

It is clear that the Intralipid contrast enhancement improves signal strength and measurement reliability in NVs in part because they are relatively small vessels with weak initial OCT signals, however, the relationship between vessel type and contrast enhancement is complex. Factors such as vessel diameter, vessel and red blood cell orientation relative to the imaging plane, and local haematocrit contribute to the apparent intensity changes following contrast injection [40,56,82]. Contrast enhancement is even heterogeneous within large vessels caused by established OCT artefacts [39], with vessel sides typically showing larger intensity changes than vessel centres due to the orientation dependent backscattering properties of red blood cells [42]. These previous studies demonstrate the importance of using contrast agents to improve the detection and measurement reliability across vessels of varying sizes and orientation, particularly for small, vertically oriented vessels such as NVs.

While the use of contrast agents with OCT clearly demonstrates increased intravascular intensity, it is also important to consider how the use of a contrast agent may impact measurements of blood flow. A previous study that also used a 3 mL/kg Intralipid bolus injection under isoflurane anaesthesia evaluated Doppler signal changes in several large pial vessels of the mouse brain following bolus injection [42]. This study found that while the vascular intensity signal increased following injection, there was no apparent change in Doppler signal. It is important that large vessels were used to evaluate potential influences on flow speed because these already provided reliable Doppler signals before the contrast injection. In our study, we do demonstrate changes in Doppler signals, but we believe this is directly related to the improvement of vessel visibility rather than changes in velocity, similar to a study by Pan et al. [55]. In their study, the authors noted increased capillary blood flow measurements using ultrahigh-resolution OCT in the mouse brain, but no significant changes in ECG or respiratory rates following a 6 mL/kg Intralipid bolus injection, twice the amount used here. Due to the increase in intensity following contrast injection, there are also apparent changes in diameter; however, we do not believe these are necessarily related to actual diameter changes. This effect seems more likely due to increased visibility of weakly scattering vessels or vessel regions, caused by orientation effects, haematocrit, and OCT artefacts as described above. Another study used an ultrahigh-resolution optical coherence microscope to visualize vessels in the mouse brain and showed apparent filling of the cell-free plasma layers at the edges of vessels after contrast agent injection, which could also give the appearance of dilating the vessels without actually causing dilation [83]. Based on the above studies, we believe that the impact on physiological responses resulting from the contrast agent injection should be minimal, but it remains an important topic for future study.

A final topic of interest is the effect of Intralipid clearance on longitudinal measurements. A previous study by Liu et al. reported an Intralipid half-life of approximately 9 minutes in rats [84], which aligned with our previous observations in mice [54], but this effect has not been rigorously studied in mice. It is a limitation of the current study that it is difficult to quantify the clearance of Intralipid from retinal images due to the influence of other factors that can degrade image quality over time, such as the development of cataracts. In the study by Pan et al., which did not have to consider the effects of cataract development, they noted a 15% decay of their measured blood flow velocity over 60 minutes following contrast injection, which they attributed to Intralipid clearance [55]. This slower decay rate may be attributable to a nonlinear relationship between tracer concentration and Doppler signal improvement. For the highest reliability, Doppler measurements should be taken as close to the contrast agent injection as possible, and in cases where longer term stability is required, continuous intravenous infusion of Intralipid could be considered.

### 4.5. Further discussions

The observed reversals in the flow direction could be attributed to several factors, including but not limited to, changes in the depth of anaesthesia, genuine physiological shifts in the flow, influence of the cardiac cycle, local retinal or choroidal pressure changes, or measurement noise. The temporal variability in flow direction changes across different NVs is an interesting observation, but the reason behind them is not completely understood. We hypothesise that this behaviour could result from various factors. These NVs form abnormal connections between the retinal and choroidal vascular networks, which increases the complexity of local hemodynamics and could create conditions where small fluctuations in the instantaneous pressure between the two networks leads to flow reversals in the individual NVs. Additionally, our acquisition was not synchronized to the cardiac cycle, and since each 3D volume acquisition took approximately 16 seconds, and the mouse heart rate is approximately 300 beats per minute (5 beats per second), our imaging captured approximately 80 cardiac cycles per volume. Different NVs therefore could have been sampled at different cardiac phases at different time points. Depending on the cardiac phase and resulting pressure changes, this has the potential to cause variability in the measured flow rates and potentially even backflow during the diastolic phase. The combination of these factors likely contributed to the flow direction changes we observed in different NVs at different times. Notably, for Mouse 1 NV2, which was anatomically isolated, all four time points showed flow values close to zero, which supports the reliability of our approach and serves as a good check for the accuracy of our flow quantification method.

The threshold adjustment from 9% to 12% for one angiogram volume (section 2.3) was necessary due to higher background noise levels in that particular volume. This elevated noise resulted from a combination of factors including the scan being acquired at 8.5 minutes post-contrast injection when contrast enhancement might have begun to diminish, and the mouse having been under imaging for approximately 14 minutes, potentially causing early cataract formation that could have potentially reduced signal quality [85]. While global thresholding generally performed well in this study, automated adaptive thresholding could reduce investigator bias and improve consistency across different imaging conditions in future applications. Nevertheless, determining the optimal adaptive segmentation method for NVs would require a careful approach, as different methods may perform differently depending on factors such as overall scan intensity, NV size and location, and contrast between NVs and surrounding retinal tissue. Investigation into these methods is of interest for future studies.

Of the six mice included in the study, mice 1, 2, 5, and 6 were anaesthetised with isoflurane, while mice 3 and 4 received ketamine/xylazine. Several studies have shown that commonly used anaesthetics, such as isoflurane and ketamine/xylazine, influence rodent ocular physiology and haemodynamics [86–89]. This could differentially affect parameters including retinal blood flow, vascular dilation, heart rate and blood pressure, which could potentially influence the measurements in our study. It is also important to note that isoflurane is known to induce vasodilation [89,90], which could potentially influence retinal blood flow measurements. The variation in anaesthesia protocols between mice in our study may therefore contribute to inter-subject variability in flow measurements. However, due to our small sample size (n = 4 for isoflurane, n = 2 for ketamine/xylazine), we cannot provide definitive assessment of anaesthesia specific effects on NV flow patterns. Further work with larger sample sizes and direct comparisons between anaesthetic regimens might clarify if there is any significant effect of the type of anaesthesia used.

One limitation of the particle tracking method presented here is the difficulty of aligning B-scans to match the orientation of NVs, so that the vessels run parallel to the scanning axis. Additionally, this method fails to capture the out of plane motion, so improper alignment or vessel tortuosity may lead to measurement inaccuracies. For future applications, implementing arbitrary scan patterns along the vessels, similar to the work done by Desissaire et al. [91], would make particle tracking more reliable and easier to implement. An additional limitation of our study is the limited scanning area of 1 mm x 1 mm, centred on the optic nerve head. Expanding the range of our scanning field of view might reveal more information on different flow patterns in the peripheral NVs. This could also help us have a more thorough grasp of the overall retinal-choroidal anastomoses flow dynamics.

While our average flow directionality approach (Fig. 5(d)) helped understand the predominant flow directionality of NVs at the animal level, it could potentially mask important temporal dynamics within individual NVs. Future studies could benefit from denser temporal sampling, with more frequent post-injection time points throughout the observation period, for better understanding the dynamic changes in NV flow patterns over time. This would enable more detailed temporal stratification analysis and potentially reveal time dependent systemic haemodynamic behaviour.

Our analysis revealed substantial variability in the directionality and magnitude of chorioretinal flow among individual mice (Fig. 5(d)). We found that only two out of six mice exhibited a strong preference for net flow into or out of choroid, while the other four mice showed net flow close to zero, indicating a balance between inflow and outflow. This suggests that in most mice, the supply and drainage of blood through these NVs was relatively balanced, and that overall directionality may not be a consistent or defining feature for this disease model. The consequence of this heterogeneity is that population-level conclusions about NV flow pattern could not be derived, and flow dynamics may need to be evaluated on a per-mouse or even per-lesion basis. This variability highlights the importance of a technique such as contrast-enhanced Doppler velocimetry, which can quantify flow in individual NVs. A further study with increased sample size and longitudinal measurements could help us better understand the full spectrum of NV haemodynamics, and to understand how these flow patterns might impact the nutrient delivery, waste removal, and disease progression in individual animals.

While our study successfully quantified blood flow in NVs with contrast-enhanced OCT, future studies could potentially benefit from incorporating adaptive optics (AO) for its enhanced lateral resolution capabilities. Implementing AO could potentially improve the velocity measurement accuracy, particularly in smaller NVs. Previous studies have shown that AO enables single-cell tracking and velocity measurements [79,92,93]. Nevertheless, implementation of adaptive optics for a small animal imaging system presents significant technical challenges, including system complexity, wavefront correction, and motion management. Future studies focusing on ultrahigh resolution flow profiles across NV diameters or analysing capillary-level anastomoses might benefit more substantially from AO integration.

Importantly, it should be noted that all our findings are based on a mouse model, and the extent to which these results translate to human neovascular AMD remain uncertain. While the VLDLR knockout mice show many features of RAP in humans, there are fundamental anatomical, physiological, and disease progression differences between mice and humans. Therefore, further research will be needed to determine how well these haemodynamic patterns and measurement approaches may apply to human pathology, and to assess the clinical relevance of our observations.

While this study focused on flow quantification and analysis, combining these measurements with other functional assessments of retinal health, e.g. electroretinography, or with structural analysis of these vessels, e.g. pericyte coverage, could provide more details about the relationship between such retinal diseases and neovascular haemodynamics. For particular applications, combining this approach with oxygen saturation measurements could provide insights into how such neovascularisations alter nutrient and oxygen delivery to the retinal tissue.

## 5. Conclusion

Using an exogenous contrast agent with OCT imaging, we characterised the flow dynamics in retinal neovascularisations in detail, revealing complex and heterogenous patterns of flow. The combination of particle tracking and Doppler OCT techniques offered complementary information, proving the reliability of our contrast enhanced OCT measurements. In conclusion, these findings not only enhance our comprehension of neovascular haemodynamics in the VLDLR knockout mouse model, but also show the potential of our contrast-enhanced OCT technique for studying such vascular pathologies in the retina. The methodologies shown in this study may find application in a wider range of such vascular disorders, potentially helping with development of therapeutic strategies to target these pathological vessels.

## Data Availability

Data underlying the results presented in this paper are not publicly available at this time but may be obtained from the authors upon reasonable request.
